# Association of early life cardiovascular risk factors with grey matter structure in young adults in the United Kingdom: the ALSPAC study

**DOI:** 10.1016/j.ebiom.2024.105490

**Published:** 2024-12-03

**Authors:** Holly T. Haines, Sana Suri, Raihaan Patel, Scott T. Chiesa

**Affiliations:** a Department of Experimental Psychology, University of Oxford, United Kingdom; b Wellcome Centre for Integrative Neuroimaging, Oxford Centre for Human Brain Activity, University of Oxford, United Kingdom; c Department of Psychiatry, Warneford Hospital, University of Oxford, United Kingdom; d Medical Research Council Unit for Lifelong Health and Ageing at UCL, Institute of Cardiovascular Science, University College London, United Kingdom

**Keywords:** Cardiovascular risk, Early life, Dementia, Grey matter structure, Obesity, Hypertension, Physical activity

## Abstract

**Background:**

Cumulative exposures to obesity, hypertension, and physical inactivity from midlife (40–65 years) onwards are three known cardiovascular risk factors for dementia and associated cerebral structural damage. Exactly how early in the lifespan sensitive periods for exposure to these risk factors begin is yet to be established, specifically with respect to onset of cerebral structural changes. We aimed to investigate whether cardiovascular risk across childhood and adolescence is already associated with cerebral structure in regions previously linked with dementia, during young adulthood.

**Methods:**

Participants were selected from the Avon Longitudinal Study of Parents and Children (ALSPAC), a UK-based prospective cohort of young people, if they had participated in a neuroimaging sub-study (N = 862). We entered data from repeated clinical assessments into mixed-effects models to estimate baseline and rate of change in body mass index (BMI) and mean arterial pressure (MAP) between ages 7–17 years, and physical activity (PA) between 11–15 years. Linear models assessed whether cardiovascular risk factors were associated with grey matter macrostructural indices (cortical thickness, surface area, volume) in young adulthood (∼20 years).

**Findings:**

BMI was found to be associated with grey matter macrostructure in nodes of Default Mode Network previously found to show atrophy in dementia. Baseline BMI was associated with thickness of precuneus cortex and entorhinal surface area, whilst rate of change in BMI across childhood and adolescence was associated with thickness of parahippocampal and middle temporal gyri and inferior parietal cortex in addition to entorhinal and parahippocampal surface area. Further, we identified associations between baseline MAP and PA and entorhinal surface area. Exploratory whole-brain analyses revealed associations between baseline and rate of change in these cardiovascular risk factors and the cortical thickness, surface area, and volume of broader groups of cortical and subcortical regions.

**Interpretation:**

Findings provide preliminary evidence that cerebral structural differences in regions linked to dementia in old age may be legacy of developmental differences associated with cardiovascular risk exposure during early life. This has relevance for lifespan models of dementia risk and timing of preventative interventions. Further work is required to generalise findings beyond this predominantly white, male, and middle-class sample to more diverse cohorts.

**Funding:**

NIHR Oxford Health BRC (NIHR203316), 10.13039/100010269Wellcome Trust (203139/Z/16/Z).

**Research in context**

**Evidence before this study**

Cumulative exposure to cardiovascular risk factors from midlife (40–65 years) onwards is associated with an increased risk for dementia and the presence of associated cerebral structural atrophy. We searched PubMed for studies of the association between cardiovascular risk and dementia-related cerebral structure in early life using combinations of search terms including: ‘cardiovascular risk’, ‘cerebral structure’, ‘dementia’, ‘childhood’, ‘adolescence’, and ‘young’. Of the studies identified, the majority reported on cohorts ranging from young adulthood to the start of midlife (18–40 years). These articles found associations between cardiovascular risk as a combined factor in addition to individual components (obesity, hypertension, and physical inactivity) and differences in cerebral structure (e.g., grey matter volumes and thickness, white matter volume and integrity) in patterns consistent with those found in older adults. Fewer studies reported investigating links between cardiovascular risk exposure and cerebral structure before young adulthood. In these limited studies, data largely consisted of a single timepoint from young people of the same age or, in limited cases, cross-sectionally across ages. Analyses also focussed predominantly on indices of overweight/obesity. These findings from early life were more mixed than those found in older cohorts, revealing age- and region-specific associations between risk exposure and cerebral structural indices. Importantly, we found no studies which explicitly investigated trajectories of cardiovascular risk exposure across early life and its association with differences in cerebral structure linked to dementia in late life.

**Added value of this study**

Our study addresses key limitations in previous work in two main ways. First, we capitalise on the extensive phenotyping obtained through multiple repeated measures in the ALSPAC cohort to investigate risk exposure (a) before adulthood, (b) longitudinally, and (c) with explicit reference to cardiovascular risk. Second, we utilise the large neuroimaging sub-studies conducted with this cohort in young adulthood to assess cerebral outcomes (a) before mid-to-late life, and (b) with consideration of regions typically associated with dementia. We found that cardiovascular risk exposure (BMI, MAP, physical inactivity) during early life may already be associated with differences in grey matter structure by the time children reach young adulthood. Importantly, these heart-brain associations were identified in several regions known to show atrophy in dementia.

**Implications of all the available evidence**

Our findings provide preliminary longitudinal evidence linking a number of highly prevalent and potentially modifiable cardiovascular risk factors in childhood and adolescence to early differences in brain structure previously shown to have known links to the development of dementia in later life. Taken together with existing research in pre-midlife cohorts, it appears that relations between cardiovascular health early in the lifespan and subsequent disease outcomes – in particular, via pathways of cumulative brain differences starting from childhood and adolescence onwards – merit greater consideration in research and clinical contexts. We encourage future investigations of early-life heart-brain links to work with diverse cohorts in order to verify generalisability of findings beyond this sample, which is predominantly white, middle-class, and male.

## Introduction

Patterns of cerebral structural damage are observed in preclinical and diagnosed dementia. Mounting evidence suggests that cumulative exposure to poor cardiovascular health is one candidate factor contributing to this damage. However, the life stage at which poor cardiovascular health poses greatest risk is yet to be established. Obesity, hypertension, and physical inactivity are three of the most widely recognised cardiovascular risk factors, included amongst the American Heart Association’s Life’s Essential Eight framework for preventing heart disease, as well as the Lancet Commission’s 12-factor lifespan model for preventing dementia.^1^^,^^2^ Lifespan models of disease adopt midlife (40–65 years) as a critical window for preventative intervention upon these risk factors, but their conclusions have been driven mainly by investigations of mid-to-late life cohorts. For instance, multiple studies have provided converging evidence that individuals with better cardiovascular health specifically during midlife show slower cognitive decline and reduced risk of dementia,^3^ while those with poorer cardiovascular health have greater cerebral damage^4^, ^5^, ^6^ and higher odds of developing dementia.^7^^,^^8^ However, relatively few studies have examined the heart-brain axis in younger cohorts, which, we propose, might prompt reconsideration of current lifespan models with respect to time-course of risk reduction and mitigation.

Preliminary evidence suggests that cumulative exposure to obesity, hypertension, and physical inactivity starting before midlife, even as early as childhood and adolescence, may predict differences in vascular and arterial health,^9^ grey matter macrostructure,^10^^,^^11^ and cognitive ability^12^ in mid-to-late life. Thus, presence of these risk factors during early life may constitute the beginnings of life-long cardiovascular risk accumulation. Given that young people during this time undergo a series of maturational changes to their body’s physiology, it is important to understand whether these early life changes in cardiovascular health might also affect concurrent cerebral outcomes.

Detectable using structural magnetic resonance imaging (MRI), notable dementia-related damage to cerebral grey matter macrostructure includes changes in *cortical thickness* (distance between the brain’s grey-white matter boundary and the pial surface); *surface area* (areal extent of grey matter on the surface of the cerebral cortex), and *volume* (product of cortical thickness and surface area).^13^, ^14^, ^15^ Variations in cortical thickness and surface area – thought to reflect neuronal density and number of cortical columns, respectively^16^ – are important indices for neuronal network structure and associated cognitive functions. A network of grey matter regions called the Default Mode Network (DMN) shows consistent abnormality in Alzheimer’s disease and other dementias.^15^ The DMN includes the anterior and posterior cingulate cortex, precuneus, and medial temporal lobe structures such as the hippocampus, parahippocampal gyrus, and entorhinal cortex. Structural and functional impairments in the DMN (such as grey matter atrophy, amyloid-beta deposition, reduced connectivity) have knock-on effects on the cognitive abilities affected in dementia (e.g., memory, thinking, and planning).^17^, ^18^, ^19^ Importantly, patterns of grey matter atrophy in DMN – especially medial temporal lobe and precuneus – are already present in preclinical phases of dementia^20^^,^^21^ and can predict transition to dementia within healthy and mild cognitive impairment groups.^22^^,^^23^

Whilst these grey matter changes are observable long before the incidence of dementia,^24^ it is currently unknown how early in the lifespan they begin and what factors contribute to them. Preliminary work in children and young adults suggests that exposure to obesity, hypertension, and physical inactivity may already be linked with concurrent grey matter macrostructure, including DMN regions, perhaps via disruption to maturational processes.^25^, ^26^, ^27^ Further work is needed to investigate possible associations between *cumulative* exposure to cardiovascular risk and cerebral structure during this key period of development.

We therefore examined whether cardiovascular profiles in childhood and adolescence were associated with grey matter macrostructure using a population-based sample of 862 young people from the Avon Longitudinal Study of Parents and Children (ALSPAC) cohort. Specifically, we assessed body mass index (BMI) and mean arterial pressure (MAP), measured repeatedly between ages 7–17 years, and level of physical activity (PA) between 11–15 years. We focus on these aspects of cardiovascular health as they are amongst the earliest emerging sources of cardiovascular health variation in early life and are the most widely recognised risk factors in current models of dementia risk.^2^ We had two key objectives: first, we assessed whether cumulative BMI, blood pressure, and PA exposures throughout childhood and adolescence were associated with grey matter thickness, surface area, and volume. Second, we assessed whether risk status (i.e., categorisation as people with hypertension, obesity, or inactivity according to cardiovascular health guidelines) stratified these associations to differentiate between changes in exposures occurring due to normal growth vs. those due to increasing risk. We performed region of interest analyses within DMN nodes as well as whole-brain exploratory analyses of grey matter macrostructure. We hypothesised that exposure to poorer cardiovascular health would be associated with poorer cerebral outcomes in the DMN and that heart-brain links would be stratified by cardiovascular risk status (i.e., individuals who do not meet recommended guidelines would drive associations between cardiovascular health and grey matter macrostructure). Preliminary evidence was found to this extent, identifying associations between characteristics of BMI, MAP, and PA during early life and later measures of grey matter thickness, surface area, and volume in several DMN nodes and exploratory whole-brain components.

## Methods

### Cohort

Data were drawn from ALSPAC, a prospective pregnancy and birth cohort based in the United Kingdom.^28^, ^29^, ^30^ Pregnant women resident in Avon, UK with expected dates of delivery between 1st April 1991 and 31st December 1992 were invited to take part in the study. 20,248 pregnancies have been identified as being eligible and the initial number of pregnancies enrolled was 14,541. Of the initial pregnancies, there was a total of 14,676 foetuses, resulting in 14,062 live births and 13,988 children who were alive at 1 year of age. When the oldest children were approximately 7 years of age, an attempt was made to bolster the initial sample with eligible cases who did not join the study originally. As a result, when considering variables collected from the age of seven onwards there are data available for more than the 14,451 pregnancies mentioned above: The number of new pregnancies not in the initial sample (known as Phase 1 enrolment) that are currently represented in the released data and reflecting enrolment status at the age of 24 is 906, resulting in an additional 913 children being enrolled. The total sample size for analyses using any data collected after the age of seven is therefore 15,447 pregnancies, resulting in 15,658 foetuses. Of these, 14,901 children were alive at 1 year of age. Further details about the cohort, study design, and sub-studies have been reported previously.^28^, ^29^, ^30^

We focussed on a subset of 862 children who took part in at least one of three multimodal MRI studies conducted whilst they were aged between 18 and 24 years: the ALSPAC-Testosterone study (N = 513, mean age = 19.6 years); the ALSPAC-Psychotic Experiences study (N = 252, mean age = 20.0 years); and the ALSPAC-Schizophrenia Recall-by-Genotype study (N = 196, mean age = 22.8 years). Within the MRI sample, 66 individuals took part in multiple sub-studies. Recruitment procedures and sub-study designs are described in the protocol paper.^30^

Please note that the study website contains details of all the data that is available through a fully searchable data dictionary and variable search tool.

### Ethics

Ethical approval for all neuroimaging sub-studies described below were obtained from the ALSPAC Ethics and Law Committee and the Local Research Ethics Committees (North Somerset & South Bristol Research Ethics Committee: 08/H0106/96). Full details of the approvals obtained are available from the study website (http://www.bristol.ac.uk/alspac/researchers/research-ethics/). Written informed consent for the use of data collected via questionnaires and clinics was obtained from participants following the recommendations of the ALSPAC Ethics and Law Committee at the time.

### Longitudinal cardiovascular risk exposures

**Fig. 1:**
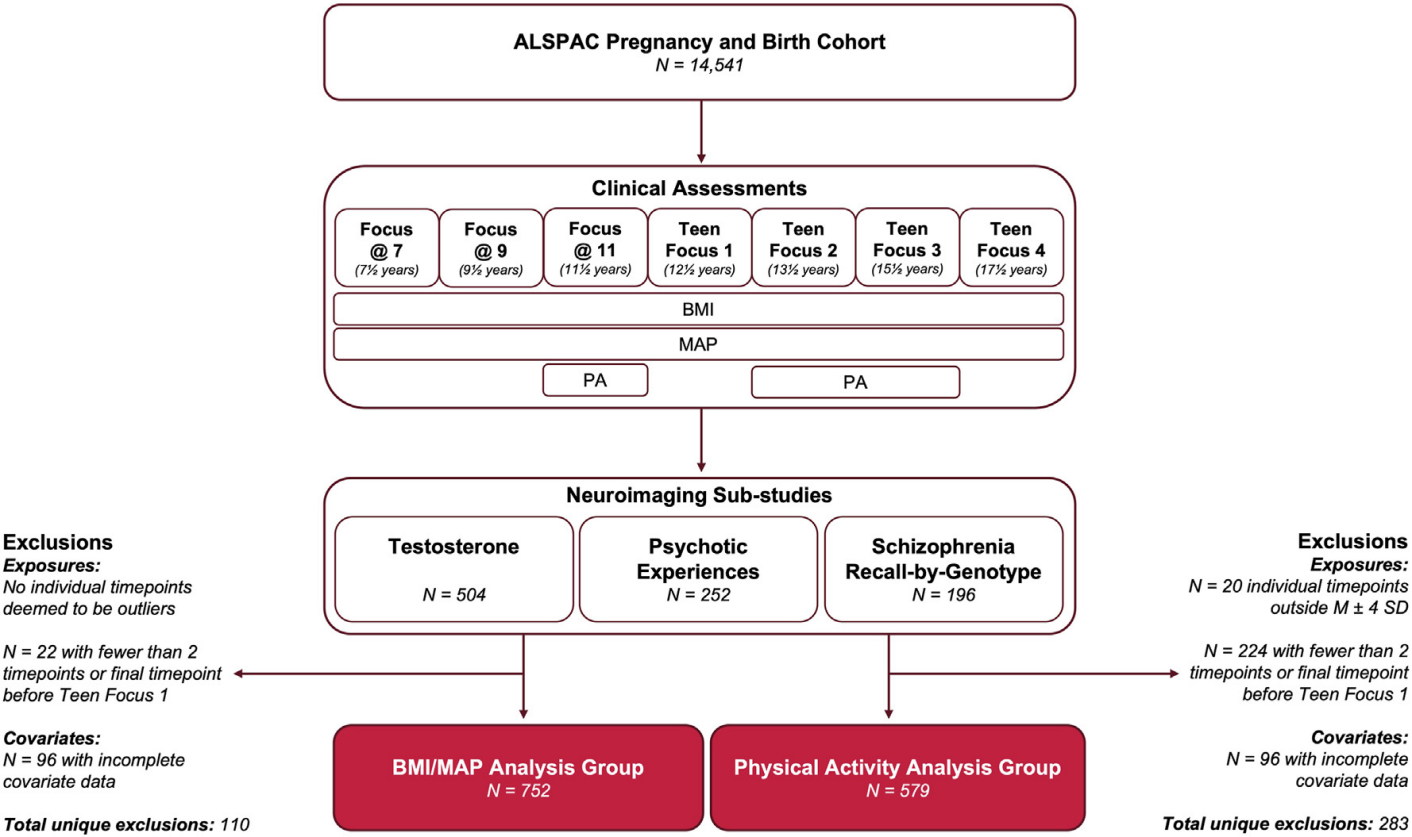
**Flowchart for sample selection and longitudinal exposures.**^a^“Total unique exclusions” refers to overall number of individuals excluded across exposure and covariate exclusion criteria, accounting for those meeting grounds for exclusion on more than one criterion. ^b^Abbreviations: BMI, body mass index; MAP, mean arterial pressure; PA, physical activity.

Study children attended clinic assessments at ages 7½ (Focus@7), 9½ (Focus@9), 11½ (Focus@11), 12½ (Teen Focus 1), 13½ (Teen Focus 2), 15½ (Teen Focus 3), and 17½ (Teen Focus 4) years. At each clinic visit, height was measured using a Harpenden Stadiometer and weight using the Tanita Body Fat Analyser (TBF 305). Body mass index (BMI) was calculated as weight (kg)/(height, m)^2^. Systolic and diastolic blood pressure were measured at rest and in a seated position. A Dinamap 9301 Vital Signs Monitor was used at the Focus@7, 9, 11 and Teen Focus 1, 3 clinics. A Dinamap 8100 Vital Signs Monitor and an Omron 705-IT was used at the Teen Focus 2 and 4 clinics, respectively. Each pressure was measured twice at each clinic and the mean of these readings was recorded. Systolic and diastolic blood pressure measurements were combined ((2 × diastolic + systolic)/3) to calculate mean arterial pressure (MAP, mmHg). Physical activity (PA) was measured at the Focus@11 and Teen Focus 2 and 3 clinics, only. Participants wore an MTI Actigraph AM7164 2.2 accelerometer on their right hip over a 7-day period. PA was defined as the average counts per minute (cpm), with moderate-to-vigorous PA operationalised as greater-or-equal to 2296 cpm. In the present study, PA measures reflect mean number of minutes per day across the 7-day assessed period for which recorded counts per minute equalled or exceeded 2296 cpm. Measurement of exposure variables is illustrated in Fig. 1.

### Neuroimaging outcomes

#### MRI acquisition

All structural MRI scans were conducted as part of a multimodal brain MRI session on the same General Electric 3T HDx scanner at the University of Cardiff, UK. Note that no scans were acquired before the final clinical assessment (Teen Focus 4). Images were acquired using a 3D fast spoiled gradient echo (FSPGR) sequence at 1 mm^3^ isotropic resolution (TR = 7.9 ms, TE = 3.0 ms, TI = 450 ms, flip angle = 20°, slice thickness = 1 mm, FOV = 256 × 192 mm, acquisition time = approximately 7 min) and were consistent across sub-studies.

#### Imaging-derived phenotypes

T1-weighted images were analysed using the FreeSurfer brain imaging software package (Version 6.0.0) by the ALSPAC team.^30^ Images were used to derive estimates of grey matter macrostructure: cortical thickness and surface area of 34 cortical regions as well as volumes of 7 subcortical regions.^30^ We averaged the values for left and right hemispheres to derive mean cortical and subcortical imaging-derived phenotypes (IDPs).

#### Regions of interest analyses

We restricted regions of interest (ROI) analyses to nodes in DMN, given its established association with dementia and midlife cardiovascular health.^6^^,^^15^ Cortical thickness and surface area analyses included eight cortical ROIs in DMN, as per^21^: caudal anterior cingulate cortex, parahippocampal gyrus, precuneus cortex, posterior cingulate cortex, inferior parietal cortex, middle temporal gyrus, entorhinal cortex, and cuneus cortex. Volumetric analyses focussed on one subcortical ROI within the DMN: hippocampus.

#### Whole-brain exploratory analyses

Whole-brain exploratory analyses included all 34 cortical regions and 7 subcortical regions. We submitted all regions to a within-modality principal components analysis (PCA) with direct oblimin rotation as a form of data reduction. Based on comparisons of eigenvalues ≥1, cumulative variance explained, and component interpretability, five components were accepted for cortical thickness (57% variance explained) and surface area (68% variance explained), and three components for volume (83% variance explained). Components were named according to the regions that loaded most strongly (Supplementary Fig. S1 and Tables S1–S3). Standardised component scores for each participant were extracted and entered as outcome variables.

### Statistics

#### Sample selection

We inspected the physiological feasibility of cardiovascular exposure datapoints lying 4 standard deviations above or below the mean at each timepoint to ensure that our final sample was representative of a full range of exposures. Under this criterion, no datapoints were excluded for BMI and MAP, whilst five PA datapoints were excluded due to suspected measurement error. Following outlier removal, we applied the following exclusion criteria. Participants were required to have data available for a minimum of two timepoints for each cardiovascular risk factor, so that longitudinal change in exposure could be estimated. We also required that the final timepoint recorded for each exposure be age 13 years (Teen Focus 2) or later. This criterion was necessary as slope estimates were intended to reflect cardiovascular risk across childhood and adolescence. Given that all three measures were expected to change considerably as children matured, slopes calculated based only on childhood data were unlikely to be representative of the full target period. Complete covariate data was also required for sample inclusion. Full exclusion pipeline for the present study is detailed in Fig. 1.

Given that there were fewer timepoints and more missing data for PA, we proceeded with two analysis groups to maximise available data: a BMI/MAP analysis group (N = 752) and a PA analysis group (N = 579). Note that almost all individuals included in the PA analysis group also met criteria for inclusion in the BMI/MAP analysis group. Suitability of sample size was evaluated a priori with reference to existing literature and verified post-hoc by assessing estimated effect size precision. Stratification by sex was not performed due to there being a relatively small number of females within analysis groups. This is largely because selection criteria for the ALSPAC-Testosterone sub-study was male sex. However, analyses controlled for effect of sex via inclusion as a covariate.

#### Slopes and intercepts of exposures

To characterise rate of change of cardiovascular measures across childhood and adolescence, we used linear mixed-effects models with natural cubic splines in RStudio version 4.2.1 using the lme4 and splines packages. For all models, age was scaled using root mean square procedures to improve model convergence due to large differences in magnitude with unscaled polynomial age terms. To facilitate interpretability of intercept estimates, scaled age terms were also centred such that a value of 0 reflected the youngest recorded age at the first clinical assessment (∼7 years for BMI and MAP; ∼11 years for PA). Natural cubic splines were used to account for potential non-linear trajectories of cardiovascular exposure over time. Model structure can be specified as follows:

Cardiovascular Exposure^a^ ∼ Spline(Age^b^, Df^c^) + (1 + (Age^b^) |Participant ID) + c

^a^ BMI, MAP, or PA.

^b^ Scaled and centred age term.

^c^ Degrees of freedom.

**Fig. 2:**
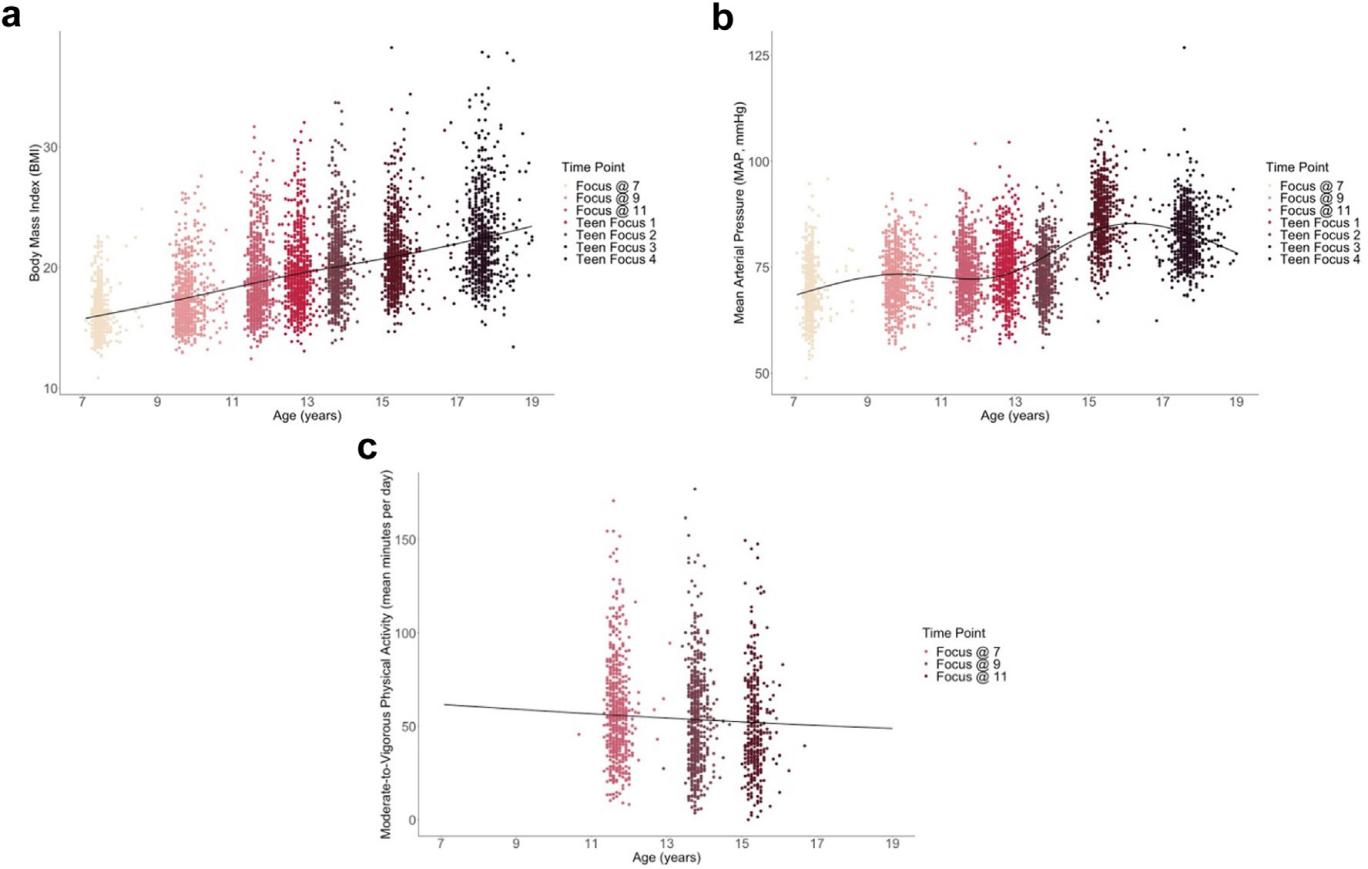
**Cardiovascular health profiles across childhood and adolescence.** Plots present change in a) BMI, b) MAP and c) PA with age at each clinical assessment during childhood and adolescence, modelled using linear mixed effects models with natural cubic splines. A 5 Df model was used for BMI, with knots at ages: 9.7, 11.8, 13.7, and 15.3 years and boundaries at: 7.1 and 19.4 years. A 4 Df model was used for MAP, with knots at ages: 9.9, 12.8, and 15.3 years and boundaries at: 7.1 and 19.4 years. A 1 Df model was used for PA, with boundaries at: 10.7 and 17.0 years. Solid lines reflect fixed effects model equation from linear mixed effects model. Sample size (n) for BMI/MAP analyses is 752, and for PA is 579.

Models with different degrees of freedom (Df) were compared for each cardiovascular exposure using goodness-of-fit metrics (A/BIC, log likelihood, Chi-squared) and visual inspection to mitigate overfitting. Inner knots were placed by default at the quantiles appropriate for each Df (quartiles for 4 Df and quintiles for 5 Df models) whilst boundary knots were positioned at youngest and oldest ages provided to each model. Exact positions of knots are given in Fig. 2. The chosen model for BMI included 5 Df as it provided better fit over the 4 Df model (*χ*^*2*^ (1, N = 752) = 4.46, *P* = 0.035), with higher Df models failing to improve fit (*χ*^2^ (1, N = 752) = 0.45, *P* = 0.50). Model selection was also supported by this model having the lowest information criterion and highest log likelihood of all models compared. We selected a 4 Df model for MAP which showed better fit over the 3 Df model (*χ*^2^ (1, N = 752) = 579.50, *P* < 0.0001) and was supported by goodness-of-fit indices. Whilst model comparisons indicated that a 5 Df model might provide a better statistical fit to our data, visual inspection of the estimated model showed signs of overfitting, especially at the tails of the distribution. Thus, the 4 Df was adopted for subsequent analyses. A 1 Df model provided best fit for PA, with a 2 Df model failing to improve fit (*χ*^2^ (1, N = 575) = 0.33, *P* = 0.57). For all participants, we extracted intercepts (representing baseline cardiovascular health) and slopes (representing rate of change across childhood and adolescence) from the mixed-effects models.

#### ROI analyses

To assess whether cardiovascular health during childhood and adolescence was associated with cerebral structure in young adulthood, we ran a series of linear models using the stats package in Rstudio. Exposure variables were the intercepts or slopes for BMI, MAP, or PA. Confounding covariates, described in detail below, were also included in linear models. Separate linear models were run for each of the three outcome modalities (cortical thickness, surface area, volume) and the nine predefined ROIs. Model specification is as follows:

Cerebral Structure^a^ ∼ Cardiovascular Exposure^b^ + Covariates + c

^a^ Surface area, cortical thickness, or volume in ROI.

^b^ Intercept or Slope for BMI, MAP, and PA.

Owing to the exploratory nature of the present study, significance results are presented without correction to highlight avenues for investigation in future work.^31^^,^^32^ Note also that reported beta estimates are unstandardised when comparing across imaging modalities.

#### Whole-brain exploratory analyses

We used linear models with the standardised PCA factor scores for cortical thickness, surface area, and volume as outcome variables. The same exposures and covariates were used as in the ROI analyses. As before, uncorrected significance values are presented for exploratory purposes.

Supplementary analyses were run using both slopes and intercepts in the same linear model to assess their unique associations with the outcome measures. The results of these analyses are presented in Supplementary Table S6 and did not differ significantly from our primary analyses.

#### Risk stratification

We assessed whether the above associations were driven by cardiovascular risk across childhood and adolescence to tease apart the effects of longitudinal changes in exposure attributable to normal growth vs. risk-related changes *beyond* normal growth. Individuals were first classified into risk groups according to published health guidelines for BMI, MAP, and PA.^1^^,^^33^ Individuals were classified as being ‘people with overweight or obesity’ if age- and sex-adjusted BMI fell within the 85th percentile or above.^33^ All those below this threshold were classified as ‘normal weight’. We did not include ‘people with underweight’ as a risk category due to the small proportion (3%) meeting relevant criterion, and because underweight is not an established cardiovascular risk factor under current AHA guidelines. Physical activity was classified using AHA recommended guidelines for adolescence.^1^ We categorised those spending 60 min or more per day participating in moderate-to-vigorous PA (i.e., ≥2296 cpm) as ‘people with high activity’ and those below this threshold as ‘people with mild activity’. Due to the small proportion of individuals (only 4%) meeting the AHA criteria for ‘people with elevated or hypertensive MAP’ (i.e., MAP ≥ the 90th percentile for those aged ≥13 years),^1^ this risk classification was not used in stratification analyses. We then computed the proportion of timepoints for which each individual was classed as ‘at risk’ to control for variability in the number of assessments attended across individuals. A threshold of 75% of recorded timepoints at risk was then adopted to classify individuals into binary risk groups, identifying children who did not meet recommended cardiovascular health criteria for the majority of the early life period studied.

We then introduced risk classification (people with obesity or mild activity) as an interaction term with cardiovascular health exposure into the linear models previously described:

Cerebral Structure^a^ ∼ Cardiovascular Exposure^b^ x Risk Group^c^ + Covariates + c

^a^ Surface area, cortical thickness, or volume.

^b^ Slope for BMI, MAP, and PA.

^c^ People with obesity vs. normal weight OR high activity vs. mild activity.

To identify associations driven by risk, we entered significant slope models into an analysis of variance (ANOVA) table to assess the evidence that effects of cardiovascular health slopes on cerebral outcomes may be equal or differ across risk groups. Results indicating the presence of interaction effects were then followed up by further examination of linear model outputs, identifying associations driven by risk as those for which the absolute magnitude of beta estimates were greater in the risk group.

#### Covariates

We selected potential confounders a priori based on relevant literature and used a directed acyclic graph to illustrate our hypothesised relationships between these variables and our exposures and outcomes of interest. Assigned sex at birth (male/female), ethnicity (white/non-white), socioeconomic status (SES; Group 1/2/3), and age at peak height velocity (aPHV) were added as covariates owing to their prior association with cardiovascular health outcomes and dementia risk.^34^, ^35^, ^36^ SES was defined as the highest parental social class based on occupation,^37^ whereby Group 1 includes Grades I-II, Group 2 includes Grades III-N/III-M, and Group 3 includes Grades IV-V. aPHV is taken as an indicator of puberty timing and its derivation has been reported previously.^38^ We further included age at MRI acquisition, intracranial volume (cortical thickness and volumetric models), hemispheric surface area (surface area models), and MRI sub-study identifier (coded according to participation in ALSPAC-Testosterone, ALSPAC-Psychotic Experiences, or ALSPAC-Schizophrenia Recall-by-Genotype).

### Role of funders

The funders played no role in study design, data analysis, or interpretation, and were not involved in the writing of the paper nor in the decision to submit the paper for publication.

## Results

### Participant demographics

Demographics of the BMI/MAP group and PA group are presented in Table 1. Across both groups, participants were predominantly white (97–98%), male (72%), and middle or upper SES (87–88%). Mean age at MRI acquisition was 20.2 years for both groups. Sub-study demographics are presented in Supplementary Tables S4 and S5.

**Table 1:** Cohort demographics for analysis groups.

Characteristics	BMI/MAP Analysis Group^a^	PA Analysis Group^a^
Age		
Age at MRI (years), *Median (IQR)*^b^	20.00 (19.3–20.7)	19.92 (19.3–20.6)
Sex		
Male	539 (72%)	415 (72%)
Female	213 (28%)	164 (28%)
Ethnicity		
White	732 (97%)	565 (98%)
Non-white	20 (3%)	14 (2%)
SES^c^		
Group 1	288 (38%)	228 (39%)
Group 2	369 (49%)	282 (49%)
Group 3	95 (13%)	69 (12%)
Sub-study identifier		
Testosterone	438 (58%)	348 (60%)
Psychotic experiences	185 (25%)	134 (23%)
Schizophrenia recall-by-genotype	129 (17%)	97 (17%)
**Total sample size, *N***	**752**	**579**

a Values reflect Number (%) unless otherwise specified.

b IQR reflects interquartile range.

c SES Group 1 includes Grades I-II, Group 2 includes Grades III-N/III-M, and Group 3 includes Grades IV-V from Office of Population Censuses and Surveys Standard Occupational Classification.

### Longitudinal change in cardiovascular exposures

In our chosen models of cardiovascular exposure, BMI showed a steady increase across childhood and adolescence (age 7.1–9.7: *β* = 3.23 [2.98, 3.32]; age 9.7–11.8: *β* = 3.97 [3.85, 4.26]; age 11.8–13.7: *β* = 5.21 [4.88, 5.38]; age 13.7–15.3: *β* = 7.9 [7.62, 8.34]; age 15.3–19.4: *β* = 7.08 [6.87, 7.48], Fig. 2a). MAP showed a non-linear increase across the same period (Fig. 2b), with periods of slower change between age 7.1–9.9 (*β* = 0.08 [−0.63, 0.78]]) and 15.3–19.4 (*β* = 5.40 [4.32, 6.47]) years and faster change between ages 9.9–12.8 (*β* = 20.26 [19.43, 21.09]), and 12.8–15.3 (*β* = 20.39 [19.02, 21.76]) years. We also noted a linear decline in moderate-to-vigorous PA across early life (*β* = −16.70 [−22.63, −10.79], Fig. 2c). The number of individuals who did not meet predefined thresholds for ideal cardiovascular health at each timepoint are given in Table 2.

**Table 2:** Descriptives for ideal cardiovascular health guidelines.

Cardiovascular risk factor^a^	Focus@ 7	Focus@9	Focus@11	Teen focus 1	Teen focus 2	Teen focus 3	Teen focus 4
Age at assessment (years), Mean (SD)	7.5 (0.2)	9.8 (0.3)	11.7 (0.2)	12.8 (0.2)	13.8 (0.2)	15.4 (0.3)	17.7 (0.3)
BMI							
Median (IQR)	15.7 (14.9–16.8)	17.0 (15.6–18.7)	18.2 (16.7–20.4)	19.0 (17.3–21.0)	19.5 (17.9–21.6)	20.5 (18.8–22.6)	21.7 (20.0–24.2)
People with Overweight/Obesity, N (%)	166 (23%)	219 (30%)	233 (32%)	217 (30%)	197 (27%)	180 (26%)	199 (28%)
Total Sample, N	721	721	731	718	717	704	708
MAP (mmHg)							
Median (IQR)	69 (65–74)	72 (68–76)	73 (70–78)	74 (70–79)	73 (70–77)	86 (81–91)	82 (78–87)
People with Elevated/Hypertensive MAP, N (%)^b^	<5 (<1%)	<5 (<1%)	<5 (<1%)	8 (1%)	<5 (<1%)	124 (18%)	27 (4%)
Total Sample, N	705	718	728	715	651	691	682
Physical activity (minutes/day)							
Median (IQR)	–	–	55 (40–73)	–	52 (34–70)	46 (32–67)	–
People with Mild Activity, N (%)	–	–	322 (59%)	–	342 (62%)	244 (68%)	–
Total Sample, N	–	–	550	–	569	361	–

a N reflects number, SD reflects standard deviation, IQR reflects interquartile range.

b Cell counts denoted ‘<5’ may include zero.

### Association of cardiovascular measures with the DMN

Table 3 summarises the results from linear models across all ROIs.

**Table 3:** Associations between cardiovascular measures and cerebral structure.

Estimate	*β*	95% CI	*P*	*Adjusted R*^*2*^
DMN ROI Analyses				
Cortical thickness				
BMI Intercept				
Precuneus	−0.01	[−0.01, −0.01]	0.013	0.04
BMI Slope				
Parahippocampal Gyrus	−0.03	[−0.05, −0.01]	0.014	0.02
Inferior Parietal Cortex	0.01	[0.01, 0.02]	0.044	0.05
Middle Temporal Gyrus	0.01	[0.01, 0.01]	0.022	0.10
Surface area				
BMI intercept				
Entorhinal cortex	−2.72	[−5.27, −0.20]	0.034	0.19
BMI slope				
Parahippocampal Gyrus	11.85	[1.59, 22.10]	0.024	0.19
Entorhinal Cortex	−10.68	[−17.58, −3.78]	0.0025	0.20
MAP intercept				
Entorhinal Cortex	−1.49	[−2.71, −0.28]	0.016	0.20
PA intercept				
Entorhinal Cortex	0.60	[0.21, 1.00]	0.0030	0.18
Whole-brain exploratory analysis				
Cortical thickness				
BMI slope				
Parietal Component	0.12	[0.01, 0.22]	0.034	0.04
Frontal Component	−0.18	[−0.28, −0.07]	0.00078	0.09
Cingulate Component	0.11	[0.01, 0.21]	0.044	0.07
MAP slope				
Occipitotemporal Component	0.22	[0.04, 0.40]	0.017	0.07
PA slope				
Parietal Component	0.09	[0.01, 0.16]	0.038	0.04
Frontal Component	0.08	[0.01, 0.16]	0.039	0.10
Surface area				
BMI intercept				
Temporal Component	−0.03	[−0.06, −0.01]	0.026	0.44
BMI slope				
Temporal Component	−0.14	[−0.22, −0.06]	0.00069	0.46
PA intercept				
Temporal Component	0.01	[0.01, 0.01]	0.010	0.42
Volume				
BMI slope				
Subcortical Motor Component	0.12	[0.06, 0.19]	0.00034	0.64

#### Cortical thickness

**Fig. 3:**
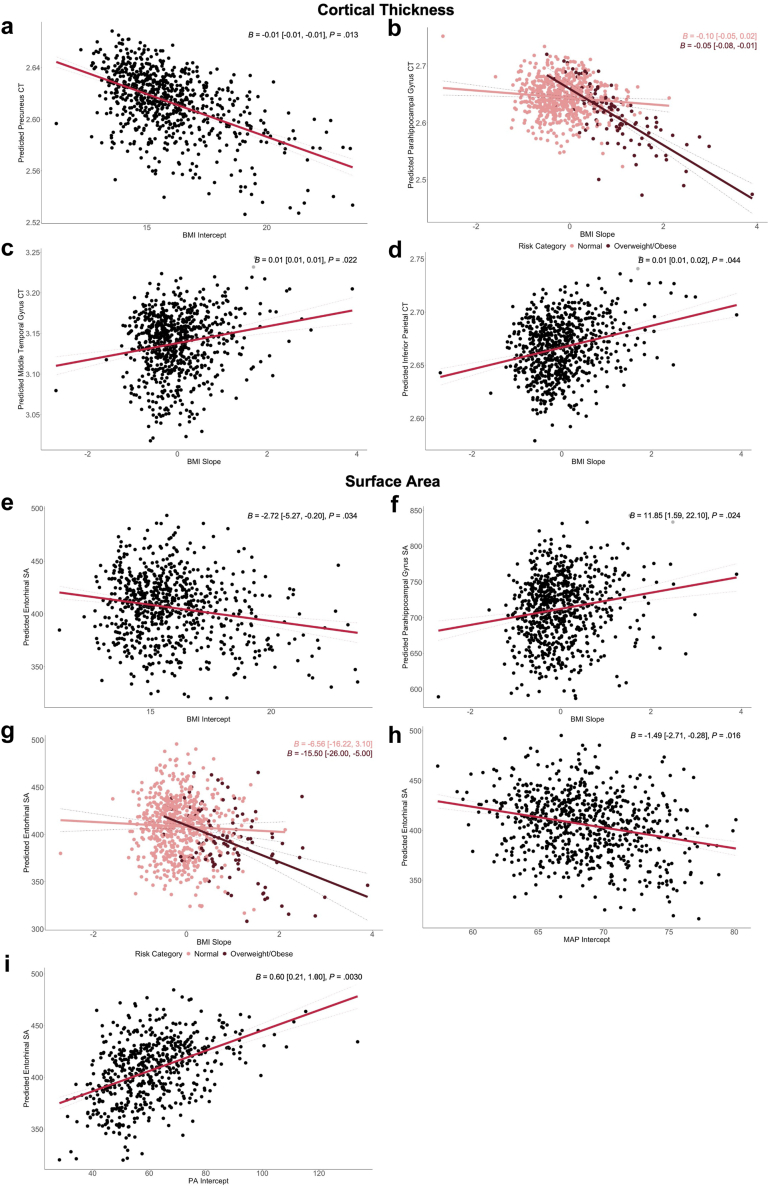
**Partial regression plots showing the associations of cardiovascular risk with cerebral structural outcomes in DMN.** Plots represent estimated marginal means for **a–d** cortical thickness and **e–i** surface area of nodes in DMN, plotted against cardiovascular slopes and intercepts. Plots **b, g** reflect risk stratification, separately plotting BMI slopes against cerebral outcomes for people with normal weight and overweight/obesity groups. Estimated marginal means are derived from linear models adjusted for MRI age, sex, ethnicity, socioeconomic status, age at Peak Height Velocity, sub-study, and relevant brain size estimate for outcome (intracranial volume for cortical thickness, volume; average hemispheric surface area for surface area). Dashed lines reflect 95% CI. Beta estimates, 95% CI, and *P*-values are reported for each association. Sample size (n) for BMI/MAP analyses is 752, and for PA is 579. ^a^Abbreviations: CT, cortical thickness; SA, surface area.

Greater BMI intercepts (representing higher baseline at ∼7 years) were associated with thinner precuneus (Fig. 3a). Moreover, faster increases in BMI across early life were associated with thinner parahippocampal gyrus and thicker inferior parietal cortex and middle temporal gyrus (Fig. 3b–d). Post-hoc analyses stratified effects by risk group (i.e., identification of individuals with overweight or obesity according to recommended health guidelines across at least 75% of recorded timepoints) to examine whether these results were driven by cardiovascular risk or developmental trends. The association between BMI slope and cortical thickness in parahippocampal gyrus was more prominent in the people with overweight/obesity group (*β* = −0.05, 95% CI [−0.08, −0.01]) than the people with normal weight group (*β* = −0.01 [−0.05, 0.02]), indicated by a difference in beta coefficients (*F* (2, 719) = 3.91, *P* = 0.020). No other results were driven by risk group. As depicted in Fig. 3b, we also identified a trend whereby individuals with greater BMI slopes (i.e., fastest rate of change in BMI across childhood and adolescence) also tended to be those classified as people with overweight or obesity over at least 75% of timepoints recorded.

Neither intercepts nor slopes for MAP and PA were associated with DMN cortical thickness in young adulthood.

#### Surface area

Greater intercepts for BMI and MAP and lower intercepts for PA all showed associations with smaller entorhinal surface area (Fig. 3e–h,i). Moreover, faster increases in BMI across early life were also associated with smaller entorhinal surface area in young adulthood in addition to larger parahippocampal gyrus surface area (Fig. 3f and g). Post-hoc analyses revealed a difference in beta estimate between risk groups (*F* (2, 719) = 5.34, *P* = 0.0050), whereby the association between BMI slopes and entorhinal cortex surface area was more pronounced in the people with overweight/obesity group (*β* = −15.50 [−26.00, −5.00]) than their people with normal weight counterparts (*β* = −6.56 [−16.22, 3.10]).

#### Volume

Cardiovascular intercepts and slopes were not associated with hippocampal volume in young adulthood.

### Association of cardiovascular measures with exploratory whole-brain components

The results from the linear models describing grey matter macrostructure as a function of cardiovascular risk are in Table 3.

#### Cortical thickness

**Fig. 4:**
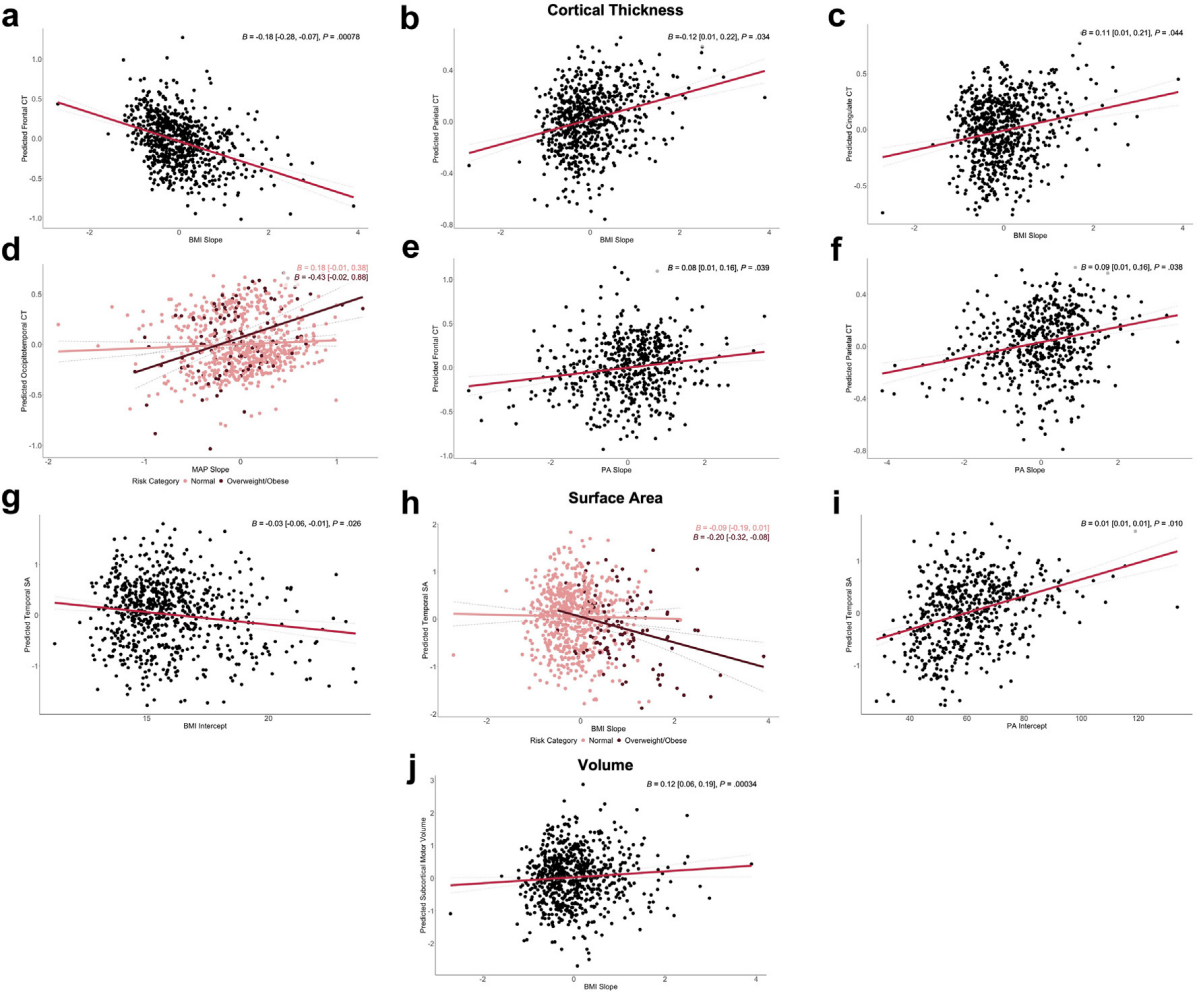
**Partial regression plot showing the association of cardiovascular risk with cerebral structural outcomes in whole-brain components.** Plots represent estimated marginal means for **a–f** cortical thickness, **g–i** surface area, **j** volume of exploratory whole-brain components, plotted against cardiovascular slopes and intercepts. Plots **d, h** reflect risk stratification, separately plotting cardiovascular slopes against cerebral outcomes for people with normal weight and overweight/obesity groups. Estimated marginal means are derived from linear regression models adjusted for MRI age, sex, ethnicity, socioeconomic status, age at Peak Height Velocity, sub-study, and relevant brain size estimate for outcome (intracranial volume for cortical thickness, volume; average hemispheric surface area for surface area). Dashed line reflects 95% CI. Beta estimates, 95% CI, and *P*-values are reported for each association. Sample size (n) for BMI/MAP analyses is 752, and for PA is 579. ^a^Abbreviations: CT, cortical thickness; SA, surface area.

Faster rate of increase in BMI in early life was associated with thinner cortex in the Frontal component (including anterior, posterior, and isthmus cingulate cortices) (Fig. 4a–c). Similarly, faster decline in PA across adolescence was associated with thinner cortex in both Frontal and Parietal components (Fig. 4e and f). Further, faster increase in MAP was associated with thicker cortex in the Occipitotemporal component (including fusiform, lingual, and parahippocampal gyri) (Fig. 4d). Stratification by risk group indicated that this result may be driven by risk (*F* (2, 719) = 3.35, *P* = 0.036), whereby this association was more pronounced in the people with overweight/obesity group ((*β* = 0.43 [−0.02, 0.88]) than the people with healthy weight group (*β* = 0.18 [−0.01, 0.38]). No other effects were driven by risk.

#### Surface area

Both greater intercepts and rate of increase for BMI in early life were associated with smaller surface area of the Temporal component (e.g., temporal pole, entorhinal cortex, inferior temporal gyrus) (Fig. 4g and h). Risk stratification analyses indicated that the association between BMI slope and Temporal surface area differed by risk group (*F* (2, 719) = 6.62, *P* = 0.0014), showing greater prominence in the people with overweight/obesity group (*β* = −0.20 [−0.32, −0.08]) than people with healthy weight individuals (*β* = −0.09 [−0.19, 0.01]). In the same Temporal component, PA intercepts were associated with larger surface area (Fig. 4i).

#### Subcortical volume

Faster increase in BMI across childhood and adolescence was linked with larger grey matter volume in the Subcortical Motor component (thalamus, caudate, putamen, and pallidum) (Fig. 4j). Cardiovascular intercepts were not associated with grey matter volume in of any of the three subcortical components.

## Discussion

In this prospective longitudinal cohort study, we present three key findings. First, BMI was shown to be an important cardiovascular exposure in early life. Baseline and rate of change in BMI showed associations with cortical thickness, surface area, and grey matter volume across DMN ROIs – often implicated in dementia later in life – and exploratory components distributed across cerebral cortex. Second, entorhinal cortex appeared to be a particularly relevant region, showing associations with BMI, MAP, and PA, consistent with its early involvement in mild cognitive impairment and dementia in older adults. Further, these effects broadly replicated in a wider component of temporal regions. Third, whole-brain exploratory analyses revealed that cortical thickness was associated with all three cardiovascular risk factors investigated across a range of components (including frontal, parietal, cingulate, and occipitotemporal regions). This may be suggestive of lifestyle-related interference in cortical thinning, known to occur during this developmental period. Overall, our results add to growing evidence that early life cardiovascular health is related to grey matter structure in young adulthood. Interesting questions arise surrounding mechanisms underlying these heart-brain associations in the young, including contributions from risk-related disruption of normal maturational processes. Taken together, this has implications for current lifespan models of dementia risk as well as for guidance on disease prevention.

Emerging literature has identified patterns of early lifestyle-related differences in cerebral structure consistent with those found in dementia. A recent cross-sectional study found that cardiovascular health in early adulthood (mean age 25 years) was associated with concurrent presence of white matter hyperintensities in the brain.^39^ Further, analyses within a different longitudinal cohort (ABCD) revealed greater decreases in grey matter volume between ages 9–11 years in children with obesity relative to normal weight counterparts.^40^ Additional cross-sectional studies have gone further, characterising early lifestyle-related variability in grey matter structure across distributed regions of cortex – including DMN nodes – and demonstrating how associations differ across developmental stages.^25^, ^26^, ^27^ We found a pattern of results linking baseline (∼7 years for BMI and MAP, ∼11 years for PA) and rate of change in cardiovascular risk exposure to cortical thickness and surface area of several DMN regions (∼20 years), as has also been found in older adults.^7^ Exploratory analyses broadened findings to components of brain regions which, together, cover much of cortex and overlap with those identified in other young cohorts.^25^^,^^26^ Overall, these findings raise the possibility that at least some cerebral structural differences observed in late-life dementia may, in fact, have their beginnings in developmental differences established in youth.^41^

One possible pathway underlying early heart-brain associations relies upon the numerous neurodevelopmental processes specific to early life and their interaction with risk exposure. In normative models of development, cortical thickness decreases non-linearly from childhood to adulthood reflecting synaptic pruning and increased myelination for adaptive neuronal circuit organisation.^42^^,^^43^ Similarly, grey matter surface area follows a slower decrease – associated with decreased gyrification of cortex.^42^ There also appears to be regional-specificity with respect to the actual time-course over which maturational change takes place.^42^^,^^44^ Importantly, neurodevelopmental processes depend on complex interactions between many biological and environmental factors. Thus, risk exposure (e.g., obesity, hypertension, physical inactivity) during this sensitive period of life may interfere with ongoing development – for example, resulting in accelerated or slower cortical thinning.^45^^,^^46^ We suggest that this may explain some of the variance in both the present study and prior early-life literature with respect to the direction of heart-brain associations (e.g., greater vs. lesser thickness, area, volume). Here, the mixed directions of associations observed in youth may be a product of region-specific timing of development and the age at which cardiovascular risks intervene on these processes.

In contrast, consistent risk-related decreases across grey matter structural indices are reported in older cohorts, despite occurring in brain regions overlapping with those implicated in early-life studies.^47^ Such differences between age groups may reflect different mechanisms underlying the observed structural differences. Risk-related deterioration in mid-to-late life grey matter macrostructure is thought to reflect active neurodegenerative processes (e.g., inflammation, amyloid-beta deposition, oxidative stress).^48^ Whilst we cannot rule out neurodegeneration as a possible explanation for our findings, we suggest that this is less likely than a developmental account of grey matter changes in the young, developing, and highly plastic brains studied in this cohort. To tease apart such accounts, future work could adopt longitudinal MRI to investigate the timing of normal maturation in studied individuals and how risk may interfere with these processes. Further, this approach would also provide helpful insight into the progression of observed structural changes, including whether they persist, worsen, or improve across adulthood.

Across ROI and exploratory analyses, BMI intercepts and, in particular, slopes were consistently associated with patterns of variation in cortical thickness and surface area. BMI is generally expected to increase during growth and development but, crucially, post-hoc investigations revealed that those showing faster increases in BMI over time also tended to be people with overweight or obesity across most recorded timepoints. Importantly, it was also these individuals, whose BMI did not meet recommended health guidelines for much of their childhood and adolescence, for whom several identified heart-brain links were most pronounced. Thus, faster risk-related increase in BMI (i.e., beyond changes expected across development) may be associated with subsequent grey matter macrostructure. Note, however, that some BMI slope effects did not appear to be driven specifically by the risk group. This could indicate a more general association between BMI and cerebral structure which is non-specific to obesity risk.^49^ Alternatively, these results may instead be a function of underpowered slope estimates in the risk group, especially given the difference in number of young people classified into each group (623 people with normal weight, 108 people with overweight/obesity) in our sample.

We found entorhinal cortex to be a key region for heart-brain associations, both as an ROI and within a component of temporal regions. This fits with theoretical frameworks identifying entorhinal cortex as a primary locus for early changes in a brain staging model for dementia.^50^ Observational research has also found consistent evidence for entorhinal atrophy in patients with subclinical dementia before evident change in other brain regions.^51^^,^^52^ Further, we note that changes to entorhinal structure were identified exclusively in surface area (and not cortical thickness). Enlarged surface area is thought to facilitate integration and differentiation between cortical columns, reflecting an efficient way for the brain to structure itself to subserve cognitive functions.^53^ Given that entorhinal cortex is a highly interconnected region playing a central role in the brain’s memory network, changes in surface area related to cardiovascular risk could subsequently have profound influence on the cognitive functions found to deteriorate in dementia.

Interestingly, we identified only weak associations between MAP and cerebral outcomes. This contrasts with our understanding of hypertension as the most consistent cardiovascular predictor of dementia and associated cerebral atrophy.^1^^,^^2^ Whilst we know that hypertensive risk in childhood is associated with changes in cardiovascular structure and function,^24^ it may be that (a) elevated MAP during early life is less relevant for *cerebral* structure in young adulthood or (b) our sample did not show sufficient variability in the upper ranges of MAP to detect any associations with elevated blood pressure. Indeed, 4 out of 7 clinical assessments reported <1% individuals with elevated or hypertensive MAP, with very few (average 7.6%) who did not meet ideal cardiovascular health thresholds in the remaining assessments. This contrasts with BMI and PA which showed greater proportions of individuals classified as people with obesity or mild activity according to published guidelines. Therefore, failure to detect associations between MAP and cerebral structure may, in fact, reflect sample constraints.

Whilst direct evidence linking pre-midlife cardiovascular risk to incidence of dementia remains to be established, the present study adds further evidence that early life may indeed appear to be a promising target in the prevention of dementia-related signatures in the brain. This supports recent calls^54^^,^^55^ for modifications to lifespan models of dementia risk to include contributions from cardiovascular health across childhood and adolescence. These models^48^^,^^55^^,^^56^ suggest that early life risk might translate to late-life disease via vascular and cerebral changes beginning in childhood and persisting through to old age that are the result of lifestyle-related differences in development. Crucially, these risk-related changes may lower one’s resilience to neurodegenerative processes later in the lifespan (termed “brain reserve”) or potentially even reflect the start of cerebral damage accumulation. Incorporating these potential pathways to disease would not only improve our understanding of dementia risk itself, but also help to inform timely prevention initiatives, intervening on relevant factors before changes begin to accumulate and/or promoting compensatory mechanisms.^57^

Moreover, our findings also align with suggestions that age-related disorders such as dementia and heart diseases may not be just diseases of *the elderly* but instead disorders of *the lifespan*, reflective of relevant health and risk factors accumulated over the course of one’s life starting from childhood. It is crucial to make this shift in perspective explicit as it could have consequences for the ways in which we conduct research (e.g., establishing more longitudinal birth cohorts, developing early-life interventions) and target public health advice.

The present study benefits from the richness of the ALSPAC dataset. Total sample size across sub-studies (N = 862) is large for brain imaging research. Moreover, repeated assessment across childhood and adolescence provides valuable opportunity to estimate variation in cardiovascular health with age as faithfully as possible – in some cases spanning 17 years between initial assessment and brain imaging.

### Limitations

Within our sample, the majority were male (72%) because selection criteria for the ALSPAC-Testosterone sub-study was male sex. Given known sex differences in cerebral structure, our findings may not be as representative of female heart-brain associations. Further, the ALSPAC cohort is predominantly white and middle class, reflective of study location (Avon, UK). As a result, the influence of some covariates (e.g., sex, ethnicity, SES) may be underestimated within our analyses. Finally, as with any observational cohort study, we cannot establish causal associations between exposures and cerebral outcomes and acknowledge possible effects of unmeasured confounding.

The present study investigated three well-established risk factors for disease in older populations (obesity, hypertension, physical inactivity) that are known to be prevalent in young cohorts. Other factors included in current lifespan models of dementia – including hyperglycaemia – may also play an important role in early-life heart-brain associations. The availability of repeat measures of glucose and insulin levels within this cohort will allow possible relationships between these risk factors and cerebral structural indices to be examined in future work. Known relationships between the cardiovascular risk factors included in the present study pose additional concerns.^58^ Indeed, whilst we examined PA and BMI separately, we cannot exclude the possibility that results for one may be in part driven by indirect effects of the other. Future work could aim to tease apart the effects of PA and BMI, acknowledging likely reciprocal relationships between them.

We note potential issues arising from systematic differences in age at MRI acquisition between sub-studies. The range between the youngest and oldest participants at time of scanning is 6½ years. In the context of brain development across young adulthood, this may reduce comparability between studies. To mitigate this where possible, we included control variables of age at MRI acquisition and sub-study identifier in all models describing cerebral outcomes. However, it remains possible that associations between cardiovascular health and cerebral structure in the present study may be obscured or underestimated by systematic differences in age, and thus developmental stage, across sub-studies. Lastly, our use of PCA with derived variables to conduct exploratory whole-brain analyses reflects somewhat arbitrary groupings of regions and different results may be found using different components. This approach also lacks granularity to identify smaller features of variation in cerebral structure related to cardiovascular risk, therefore future work conducting voxel-wise analyses is needed.

In this longitudinal study, estimated baseline and rate of change in BMI, MAP, and PA across childhood and adolescence were associated with grey matter macrostructure in young adulthood, particularly in brain regions affected in Alzheimer’s disease and dementia. This adds to growing evidence that, at least some, cerebral structural changes linked with dementia in old age may be developmental in nature and have persisted since young adulthood. This study supports proposals to reconsider current lifespan models of dementia risk and include contributions from early life cardiovascular health to brain reserve and cumulative burden over the life course. Further research is required to verify these findings and is encouraged to investigate additional structural modalities (e.g., grey matter density and white matter integrity and microstructure) to establish a more complete picture of how early cardiovascular health relates to cerebral structure in young adulthood.

## Contributors

SS and SC conceptualised the study. HH performed statistical analyses under the supervision of SS. RP and SC advised on methodologies and data analysis. HH and SS drafted the initial manuscript. All authors contributed to the review and editing of the final manuscript and have read and approved the final version. HH and SC have full access to and have verified the underlying data.

## Data sharing statement

The informed consent obtained from ALSPAC participants does not allow the data to be made freely available through any third party maintained public repository. However, data used for this submission can be made available on request to the ALSPAC Executive. The ALSPAC data management plan describes in detail the policy regarding data sharing, which is through a system of managed open access. Full instructions for applying for data access can be found here: http://www.bristol.ac.uk/alspac/researchers/access/. The ALSPAC study website contains details of all the data that are available (http://www.bristol.ac.uk/alspac/researchers/our-data/).

## Declaration of interests

We declare no competing interests.
