# Supplementary material for: Association of early life cardiovascular risk factors with grey matter structure in young adults in the United Kingdom: the ALSPAC study
Source: eBioMedicine. 2024 Dec 3;110:105490. doi: 10.1016/j.ebiom.2024.105490 (PMC11652839; doi:10.1016/j.ebiom.2024.105490)
Supplement: Supplementary Figures S1 and Tables S1–S6 [file mmc1.docx]

**Supplementary Materials**

Supplementary Figures 2

Supplementary Figure 1 2

Supplementary Tables 3

Supplementary Table 1 3

Supplementary Table 2 5

Supplementary Table 3 7

Supplementary Table 4 8

Supplementary Table 5 9

Supplementary Table 6 10

# Supplementary Figures

Supplementary Figure 1: *Scree Plots for Within-modality PCA with Direct Oblimin Rotation for Cortical Thickness, Surface Area, and Volume*


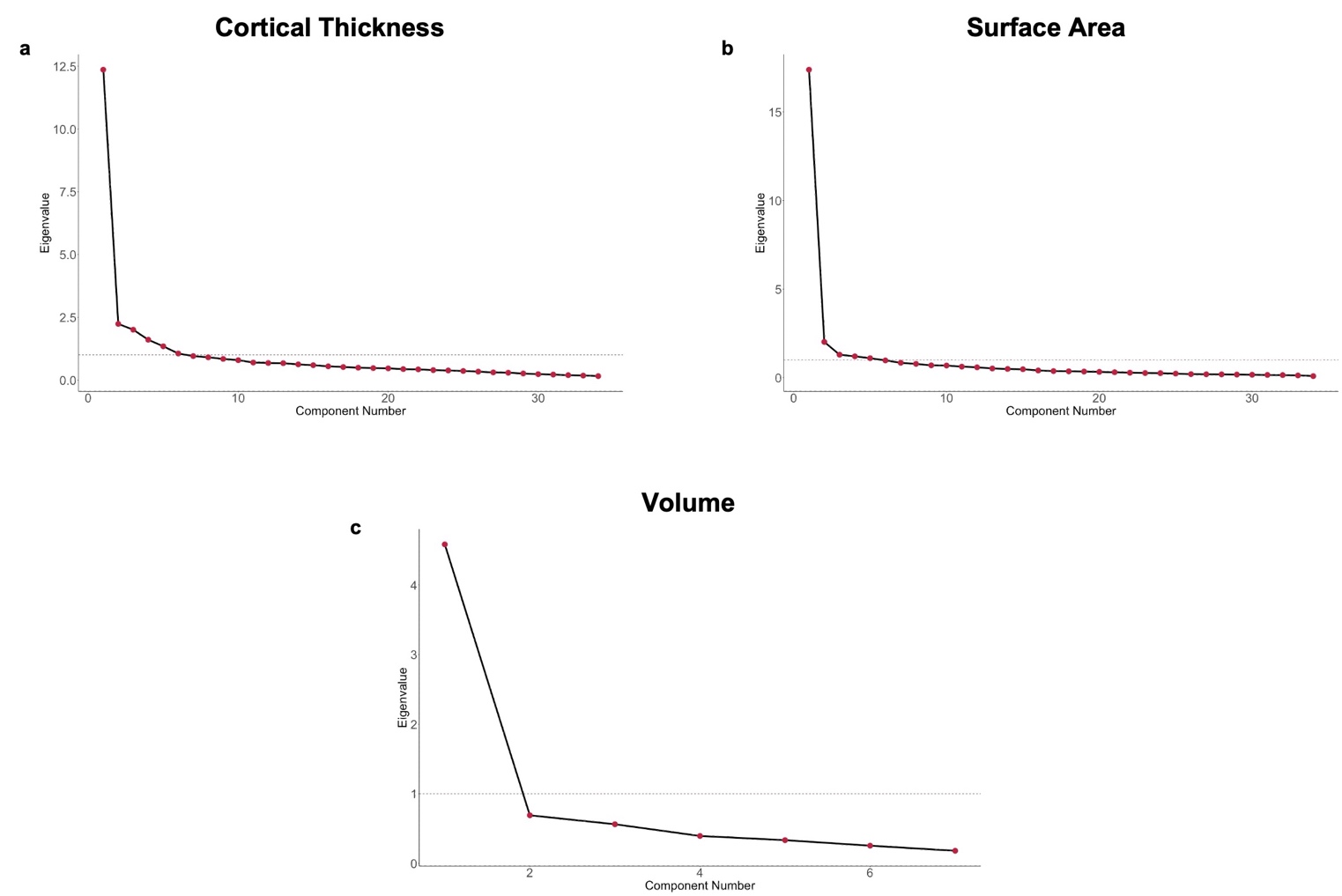


Plots reflect scree plots for **a** cortical thickness, **b** surface area, **c** volume within-modality PCA with direct Oblimin rotation. Dashed line reflects Eigenvalue of 1, providing one indicator for adequacy of component number. Five components were selected for cortical thickness and surface area, explaining 57% and 68% of the variance, respectively. Three components were selected for volume, explaining 83% of variance.

# Supplementary Tables

Supplementary Table 1: *Component Loadings by Cortical Region for Within-modality PCA with Direct Oblimin Rotation for Cortical Thickness*

|  | **Cortical Thickness** | | | | |
| --- | --- | --- | --- | --- | --- |
|  | **Parietal Component** | **Frontal Component** | **Occipitotemporal Component** | **Temporal Component** | **Cingulate Component** |
| Superior Parietal Cortex | 0.88 | -0.04 | -0.01 | -0.06 | 0.11 |
| Paracentral Lobule | 0.83 | 0.01 | -0.04 | -0.04 | -0.05 |
| Precentral Gyrus | 0.77 | 0.07 | 0.03 | 0.10 | -0.19 |
| Inferior Parietal Cortex | 0.75 | 0.07 | -0.02 | 0.03 | 0.16 |
| Postcentral Gyrus | 0.73 | -0.08 | 0.15 | -0.01 | -0.08 |
| Supramarginal Gyrus | 0.71 | 0.06 | 0.03 | 0.16 | 0.11 |
| Precuneus Cortex | 0.67 | 0.06 | 0.05 | 0.01 | 0.22 |
| Caudal Middle Frontal Gyrus | 0.67 | 0.35 | 0.00 | 0.02 | -0.22 |
| Lateral Occipital Cortex | 0.62 | -0.04 | 0.21 | -0.04 | 0.25 |
| Middle Orbitofrontal Cortex | -0.20 | 0.80 | 0.06 | -0.08 | 0.12 |
| Lateral Orbital Frontal Cortex | 0.04 | 0.67 | 0.05 | 0.18 | 0.07 |
| Rostral Middle Frontal Gyrus | 0.39 | 0.66 | 0.01 | -0.03 | -0.07 |
| Rostral Anterior Cingulate Cortex | -0.15 | 0.64 | 0.03 | 0.07 | 0.22 |
| Frontal Pole | 0.05 | 0.64 | 0.05 | -0.16 | 0.02 |
| Pars Orbitalis | 0.18 | 0.56 | 0.07 | 0.14 | -0.09 |
| Pars Triangularis | 0.31 | 0.49 | -0.02 | 0.11 | 0.10 |
| Superior Frontal Gyrus | 0.57 | 0.49 | -0.04 | 0.02 | -0.03 |
| Pars Opercularis | 0.29 | 0.45 | -0.04 | 0.26 | 0.03 |
| Lingual Gyrus | 0.03 | 0.07 | 0.81 | -0.15 | -0.09 |
| Fusiform Gyrus | 0.08 | 0.04 | 0.70 | 0.27 | -0.09 |
| Parahippocampal Gyrus | -0.21 | 0.02 | 0.66 | 0.17 | 0.06 |
| Temporal Pole | -0.03 | 0.04 | 0.17 | 0.56 | 0.11 |
| Superior Temporal Gyrus | 0.39 | 0.00 | 0.12 | 0.53 | 0.20 |
| Inferior Temporal Gyrus | 0.23 | 0.17 | 0.27 | 0.40 | 0.07 |
| Entorhinal Cortex | -0.06 | 0.02 | 0.43 | 0.40 | -0.02 |
| Middle Temporal Gyrus | 0.45 | 0.14 | 0.07 | 0.39 | 0.15 |
| Insular Cortex | 0.08 | 0.12 | 0.09 | 0.35 | 0.37 |
| Superior Temporal Sulcus | 0.50 | -0.03 | -0.09 | 0.35 | 0.12 |
| Transverse Temporal Cortex | 0.42 | -0.14 | 0.17 | 0.30 | 0.06 |
| Cuneus Cortex | 0.48 | -0.05 | 0.31 | -0.32 | 0.31 |
| Pericalcarine Cortex | 0.20 | 0.00 | 0.49 | -0.46 | 0.22 |
| Isthmus Cingulate Cortex | 0.01 | 0.10 | 0.05 | 0.05 | 0.63 |
| Posterior Cingulate Cortex | 0.23 | 0.08 | -0.21 | 0.13 | 0.61 |
| Caudal Anterior Cingulate Cortex | -0.11 | 0.31 | -0.03 | -0.04 | 0.59 |

PCA produced five components for cortical thickness (57% variance explained). Component loadings are from pattern matrix with direct oblimin rotation.

Supplementary Table 2: *Component Loadings by Cortical Region for Within-modality PCA with Direct Oblimin Rotation for Surface Area*

|  | **Surface Area** | | | | | |
| --- | --- | --- | --- | --- | --- | --- |
|  | **Temporoparietal Component** | **Occipital Component** | **Temporal Component** | **Cingulate Component** | **Frontotemporal Component** |  |
| Superior Parietal Cortex | 0.38 | 0.23 | 0.07 | 0.26 | 0.12 |  |
| Paracentral Lobule | 0.27 | -0.02 | 0.05 | 0.31 | 0.34 |  |
| Precentral Gyrus | 0.32 | 0.16 | 0.16 | 0.18 | 0.30 |  |
| Inferior Parietal Cortex | 0.42 | 0.07 | 0.14 | 0.49 | -0.11 |  |
| Postcentral Gyrus | 0.39 | 0.15 | 0.15 | 0.26 | 0.22 |  |
| Supramarginal Gyrus | 0.45 | 0.04 | 0.09 | 0.19 | 0.30 |  |
| Precuneus Cortex | 0.34 | 0.18 | 0.12 | 0.29 | 0.17 |  |
| Caudal Middle Frontal Gyrus | 0.26 | 0.13 | 0.13 | 0.32 | 0.11 |  |
| Lateral Occipital Cortex | 0.18 | 0.66 | 0.15 | -0.04 | 0.09 |  |
| Middle Orbitofrontal Cortex | -0.03 | 0.04 | 0.30 | 0.44 | 0.28 |  |
| Lateral Orbital Frontal Cortex | -0.03 | 0.04 | 0.25 | 0.36 | 0.42 |  |
| Rostral Middle Frontal Gyrus | 0.02 | 0.16 | 0.17 | 0.43 | 0.31 |  |
| Rostral Anterior Cingulate Cortex | -0.08 | 0.06 | 0.15 | 0.71 | 0.13 |  |
| Frontal Pole | -0.04 | 0.14 | 0.28 | 0.13 | 0.31 |  |
| Pars Orbitalis | -0.13 | 0.13 | 0.16 | 0.21 | 0.55 |  |
| Pars Triangularis | -0.20 | 0.09 | -0.01 | 0.00 | 0.87 |  |
| Superior Frontal Gyrus | 0.16 | 0.03 | 0.23 | 0.30 | 0.41 |  |
| Pars Opercularis | 0.07 | 0.06 | -0.07 | 0.01 | 0.74 |  |
| Lingual Gyrus | 0.10 | 0.86 | -0.01 | 0.06 | -0.05 |  |
| Fusiform Gyrus | 0.43 | 0.22 | 0.34 | -0.01 | 0.18 |  |
| Parahippocampal Gyrus | 0.70 | 0.11 | -0.01 | -0.27 | 0.10 |  |
| Temporal Pole | -0.11 | 0.08 | 0.87 | 0.00 | -0.05 |  |
| Superior Temporal Gyrus | 0.34 | 0.03 | 0.07 | 0.16 | 0.51 |  |
| Inferior Temporal Gyrus | 0.30 | 0.12 | 0.48 | 0.24 | -0.02 |  |
| Entorhinal Cortex | 0.01 | -0.09 | 0.91 | -0.11 | -0.01 |  |
| Middle Temporal Gyrus | 0.41 | 0.07 | 0.17 | 0.41 | 0.06 |  |
| Insular Cortex | 0.26 | 0.06 | 0.11 | 0.09 | 0.53 |  |
| Superior Temporal Sulcus | 0.50 | -0.14 | 0.01 | 0.44 | 0.06 |  |
| Transverse Temporal Cortex | 0.22 | -0.05 | -0.02 | -0.09 | 0.75 |  |
| Cuneus Cortex | -0.08 | 0.85 | 0.02 | -0.01 | 0.11 |  |
| Pericalcarine Cortex | -0.10 | 0.99 | -0.06 | -0.06 | -0.05 |  |
| Isthmus Cingulate Cortex | 0.16 | 0.48 | 0.03 | 0.33 | -0.01 |  |
| Posterior Cingulate Cortex | 0.10 | 0.10 | -0.12 | 0.68 | 0.16 |  |
| Caudal Anterior Cingulate Cortex | -0.10 | 0.05 | -0.03 | 0.86 | -0.01 |  |

PCA produced five components for surface area (68% variance explained). Component loadings are from pattern matrix with direct oblimin rotation.

Supplementary Table 3: *Component Loadings by Subcortical Region for Within-modality PCA with Direct Oblimin Rotation for Volume*

|  | | **Volume** | | | | |  |
| --- | --- | --- | --- | --- | --- | --- | --- |
|  | | **Subcortical Motor Component** | | **Limbic Component** | | **Accumbens Component** | |
| Pallidum | 0.90 | | 0.09 | | -0.09 | |  |
| Caudate | 0.90 | | -0.17 | | 0.13 | |  |
| Putamen | 0.80 | | 0.07 | | 0.05 | |  |
| Thalamus | 0.67 | | 0.29 | | -0.01 | |  |
| Hippocampus | -0.02 | | 0.93 | | 0.05 | |  |
| Amygdala | 0.10 | | 0.83 | | 0.03 | |  |
| Accumbens | 0.01 | | 0.04 | | 0.98 | |  |

PCA produced three components for volume (83% variance explained). Component loadings are from pattern matrix with direct oblimin rotation.

Supplementary Table 4: *Cohort Demographics for the ALSPAC neuroimaging sub-studies.*

| **Characteristics** | **ALSPAC-Testosterone** | **ALSPAC-PE** | **ALSPAC-SCZ-RbG** |
| --- | --- | --- | --- |
| **Age (years)** |  |  |  |
| Age at MRI, *Mean (SD)* | 19.58 (0.82) | 20.02 (0.52) | 22.73 (0.76) |
| **Sex** |  |  |  |
| Male | 439 (100%) | 59 (31%) | 47 (35%) |
| Female | 0 (0%) | 133 (69%) | 88 (65%) |
| **Ethnicity** |  |  |  |
| White | 427 (97%) | 184 (96%) | 135 (100%) |
| Non-white | 12 (3%) | 8 (4%) | 0 (0%) |
| **SES** |  |  |  |
| Group 1 | 173 (40%) | 65 (34%) | 56 (41%) |
| Group 2 | 217 (49%) | 99 (52%) | 58 (43%) |
| Group 3 | 49 (11%) | 28 (14%) | 21 (16%) |
| **Total Sample Size, *N*** | 439 | 192 | 135 |

^a^Values reflect number (%), unless otherwise specified.

^b^Data are presented for participants with complete covariate data.

Supplementary Table 5: *Grey Matter Macrostructure in DMN Regions for the ALSPAC neuroimaging sub-studies.*

| **Region** | **ALSPAC-Testosterone** | **ALSPAC-PE** | **ALSPAC-SCZ-RbG** |
| --- | --- | --- | --- |
| **Cortical Thickness (mm)** | | | |
| Parahippocampal Gyrus | 2.63 (0.21) | 2.66 (0.20) | 2.65 (0.20) |
| Precuneus Cortex | 2.61 (0.11) | 2.62 (0.10) | 2.58 (0.12) |
| Posterior Cingulate Cortex | 2.99 (0.14) | 2.90 (0.13) | 2.88 (0.12) |
| Inferior Parietal Cortex | 2.67 (0.11) | 2.68 (0.10) | 2.65 (0.10) |
| Middle Temporal Gyrus | 3.16 (0.11) | 3.13 (0.11) | 3.09 (0.12) |
| Entorhinal Cortex | 3.13 (0.25) | 3.37 (0.23) | 3.34 (0.25) |
| Anterior Cingulate Cortex | 3.02 (0.21) | 3.03 (0.23) | 3.01 (0.20) |
| Cuneus Cortex | 2.07 (0.13) | 2.11 (0.13) | 2.08 (0.12) |
| **Surface Area (mm^2^)** | | | |
| Parahippocampal Gyrus | 732.42 (103.59) | 685.65 (98.18) | 683.03 (88.19) |
| Precuneus Cortex | 4094.34 (478.38) | 3779.80 (494.10) | 3727.41 (491.83) |
| Posterior Cingulate Cortex | 1097.97 (134.62) | 1053.60 (141.34) | 1010.24 (122.34) |
| Inferior Parietal Cortex | 5151.77 (600.18) | 4705.83 (613.95) | 4684.95 (589.19) |
| Middle Temporal Gyrus | 3647.37 (385.94) | 3287.59 (402.14) | 3273.78 (413.93) |
| Entorhinal Cortex | 417.88 (69.51) | 398.26 (69.08) | 389.83 (60.20) |
| Anterior Cingulate Cortex | 672.20 (119.55) | 635.66 (113.72) | 611.75 (105.81) |
| Cuneus Cortex | 1579.28 (211.75) | 1478.54 (209.56) | 1468.57 (186.72) |
| **Volume (mm^3)^** | | | |
| Hippocampus | 4733.10 (299.68) | 4291.29 (429.22) | 4420.94 (423.11) |

^a^Values reflect Mean (SD), unless otherwise specified.

^b^Data are presented for participants with complete covariate data.

Supplementary Table 6: *Combined Slope and Intercept Linear Model Outputs*

| **Estimate** | **Intercept** | | **Slope** | | **Adjusted *R^2^*** |
| --- | --- | --- | --- | --- | --- |
|  | ***β*** | ***P*** | ***β*** | ***P*** |  |
| **DMN ROI Analyses** | | | | | |
| Cortical Thickness | | | | | |
| **BMI** |  |  |  |  |  |
| Parahippocampal Gyrus | -0.01  [-0.01, -0.01] | .60 | -0.03  [-0.05, -0.01] | .036 | .02 |
| Precuneus | 0.01  [-0.01, -.01] | .0072 | 0.01  [-0.01, 0.02] | .34 | .03 |
| Inferior Parietal Cortex | -0.01  [-0.01. -0.01] | .035 | 0.02  [0.01, 0.03] | .0095 | .04 |
| Middle Temporal Gyrus | -0.01  [-0.01, 0.01] | .39 | 0.02  [0.01, 0.03] | .018 | .07 |
| Surface Area | | | | | |
| **BMI** |  |  |  |  |  |
| Parahippocampal Gyrus | -0.31  [-4.33, 3.72] | .88 | 12.15  [1.15, 23.15] | .030 | .19 |
| Entorhinal Cortex | -1.53  [-4.24, 1.18] | .27 | -9.18  [-16.57, -1.79] | .015 | .20 |
| **MAP** |  |  |  |  |  |
| Entorhinal Cortex | -1.69  [-3.02, -0.26] | .013 | -4.60  [-17.32, 8.12] | .48 | .19 |
| **PA** |  |  |  |  |  |
| Entorhinal Cortex | 0.57  [0.11, 1.03] | .016 | -0.90  [-6.89, 5.08] | .77 | .18 |
| **Exploratory Whole-brain Analyses** | | | | | |
| Cortical Thickness | | | | | |
| **BMI** |  |  |  |  |  |
| Parietal | -0.03  [-0.08, 0.01] | .11 | 0.15  [0.03, 0.27] | .01 | .03 |
| Frontal | 0.01  [-0.03, 0.05] | .53 | -0.19  [-0.29, -0.08] | .00053 | .16 |
| Cingulate | -0.05  [-0.09, -0.01] | .03 | 0.16  [0.04, 0.27] | .0067 | .09 |
| **MAP** |  |  |  |  |  |
| Occipitotemporal | -0.01  [-0.03, 0.01] | .35 | 0.19  [-0.01, 0.39] | .065 | .06 |
| **PA** |  |  |  |  |  |
| Parietal | -0.01  [-0.01, 0.01] | .90 | 0.09  [-0.01, 0.18] | .074 | .03 |
| Frontal | -0.01  [-0.01, 0.01] | .21 | 0.04  [-0.04, 0.13] | .35 | .16 |
| Surface Area | | | | | |
| **BMI** |  |  |  |  |  |
| Temporal | -0.02  [-0.05, 0.01] | .28 | -0.12  [-0.21, -0.04] | .0054 | .45 |
| **PA** |  |  |  |  |  |
| Temporal | 0.01  [0.00, 0.01] | .048 | -0.02  [-0.09, 0.05] | .012 | .42 |
| Volume | | | | | |
| **BMI** |  |  |  |  |  |
| Subcortical Motor | 0.01  [-0.01, 0.01] | .87 | 0.12 [0.05, 0.19] | .0010 | .64 |
|  |  |  |  |  |  |

^a^Beta column reflects beta-estimate [95% confidence intervals]
